# Impact of Ketamine and Propofol on Cognitive Function in Elderly Patients: A Systematic Review

**DOI:** 10.7759/cureus.79091

**Published:** 2025-02-16

**Authors:** Gilles Van de Vel, Sojeong Mun, Shahab Ud Din Zia, Roopa Chalasani, Pranav S Shukla, Iana Malasevskaia

**Affiliations:** 1 Hospital Medicine, King's Mill Hospital, Mansfield, GBR; 2 Physical Medicine and Rehabilitation, Hallym University College of Medicine, Chuncheon, KOR; 3 Medicine and Surgery, Pak International Medical College, Peshawar, PAK; 4 Research, Wake Forest Institute for Regenerative Medicine, Winston Salem, USA; 5 General Practice, Grant Medical College and Sir JJ Group of Hospitals, Mumbai, IND; 6 Obstetrics and Gynecology, Private Clinic 'Yana Alexandr', Sana'a, YEM

**Keywords:** delirium, elderly, ketamine, postoperative cognitive dysfunction, propofol

## Abstract

Anesthesia has been thought to impact cognitive function in the elderly, although the exact pathophysiology remains uncertain. This systematic review aimed to analyze the impact of ketamine and propofol on cognitive function in elderly patients. Following the Preferred Reporting Items for Systematic Reviews and Meta-Analyses (PRISMA) guidelines, a comprehensive search was conducted across PubMed/Medline, Cochrane Central Register of Controlled Trials (CENTRAL), Europe PMC, ScienceDirect, ClinicalTrials.gov, and EBSCO Open Dissertations on November 17, 2024. After screening, the methodological quality of the included studies was assessed using the Cochrane Risk-of-Bias-2 Tool and the Newcastle Ottawa Scale. Studies were included if they focused on patients aged 60 and older, encompassing 3,149 participants across 19 studies, predominantly randomized controlled trials. Key outcomes assessed included postoperative cognitive dysfunction (POCD) and postoperative delirium (POD). Results indicated that many studies found no significant differences in cognitive outcomes between certain anesthetic drugs. However, ketamine was likely associated with an increased risk of POCD, similar to propofol, when compared to remimazolam and dexmedetomidine. Notably, ketofol reduced POD incidence compared to placebo, while higher propofol doses were linked to an increased incidence, and severity of hypoactive POD. The most evident finding was that propofol attenuated POCD compared to inhaled anesthetic agents. Given this, it is crucial for clinicians to carefully consider anesthetic choices for elderly patients. Future research should focus on larger multicenter trials to further validate these results and explore the long-term cognitive effects of various anesthetic agents.

## Introduction and background

Postoperative cognitive dysfunction (POCD) is characterized by a decline in neurocognitive function following anesthesia and surgical procedures [1]. POCD presents in various ways, including postoperative delirium (POD), which is marked by acute confusion within the first 72 hours post-surgery [1]. While many patients return to their baseline, the long-term consequences can be profound, such as an increased mortality risk and prolonged hospital stays, alongside a 10-fold increase in the risk of developing dementia [2].

A study published in 2020 examined 2,380,473 patients across 4,285 hospitals, revealing that 44,974 patients (1.9%) developed POCD. This retrospective cohort study highlighted an average increase of $17,275 in healthcare costs in the subsequent year per individual [3]. Additionally, a review of more than 46 million discharge records demonstrated a significant rise in the incidence of POCD correlating with advancing age, particularly among the elderly [2].

The United Nations defines an "older person" as anyone aged 60 years or older [4]. According to the World Health Organization (WHO), this demographic comprised one billion individuals in 2019, with projections indicating a rise to over two billion by 2050 [5]. Notably, the mean age of surgical patients has tremendously increased, rising from 47.5 years in England in 1999 to 54.2 years in 2015, while American forecasts predict an average surgical age of 57.7 years by 2030 [6,7]. As the elderly population grows, healthcare systems must adapt to address their specific needs and manage associated costs [8]. This evolving landscape underscores the necessity for a thorough understanding of how anesthesia factors into postoperative cognitive outcomes.

Among the various anesthetic agents, ketamine and propofol are commonly employed for both induction and maintenance of anesthesia. These agents exhibit distinct pharmacological profiles that may influence cognitive function [9,10]. Ketamine, a noncompetitive N-methyl-D-aspartate (NMDA) receptor antagonist, possesses anti-inflammatory properties that can reduce postoperative inflammatory markers [11]. This reduction in neuroinflammation may, in turn, mitigate POCD [12]. In contrast, propofol is thought to provide central nervous system protection by attenuating oxidative stress [13]. While some studies suggest that both ketamine and propofol may lower POCD rates [14-16], others report conflicting findings [12,17,18] or indicate that the clinical significance is negligible [19,20]. A recent investigation explored the combined effects of these agents, referred to as "ketofol," on cognitive outcomes and showed a statistically significant reduction in POCD [13].

Despite the critical importance of understanding the impact of these agents on cognitive function, there remains a paucity of comparative studies specifically targeting the elderly population. Therefore, elucidating the differential impacts of ketamine and propofol, as well as their combined effects, on cognitive function in older patients is essential for optimizing anesthesia protocols and enhancing postoperative recovery. This systematic review aims to evaluate and compare the influence of ketamine and propofol on cognitive function in elderly patients receiving anesthesia.

## Review

Methods

Study Design

This systematic review followed the Preferred Reporting Items for Systematic Reviews and Meta-Analyses (PRISMA) 2020 guidelines [21] and investigated the impact of ketamine and/or propofol on cognitive function in the elderly population, specifically focusing on patients aged 60 years and older who underwent anesthesia. The research question guiding this review was: How did ketamine and/or propofol influence cognitive function in elderly patients undergoing anesthesia? This inquiry was framed within the PICO framework, where the patient population consisted of elderly patients aged 60 years and older, the intervention involved the administration of ketamine and/or propofol during anesthesia, the comparison included the effects of ketamine versus propofol, and the outcomes were measurable cognitive function assessments using validated tools such as the Mini-Mental State Examination (MMSE) and/or the Montreal Cognitive Assessment (MoCA).

Eligibility Criteria

The eligibility criteria for this review were clearly defined. Inclusion criteria specified only original research studies would be considered, including randomized controlled trials (RCTs), controlled clinical trials, and observational studies such as cohort, case-control, and cross-sectional studies. The population of interest was elderly patients, defined as those aged 60 and above. Studies needed to compare the effects of ketamine and/or propofol on cognitive function, with outcomes measured using validated cognitive assessment tools post-intervention. Intervention could be general anesthesia or regional anesthesia with or without adjunctive sedation. Furthermore, only studies published in English were included, with no restriction on the publication date.

Conversely, the exclusion criteria focused on non-original research, such as reviews, meta-analyses, case reports, abstracts, commentaries, and editorials. Trials that were incomplete or those that had been completed without results available were also excluded. Additionally, studies involving younger populations (under 60 years) or those with pre-existing cognitive impairments unrelated to anesthesia were not considered. Studies that did not specifically assess ketamine or propofol as primary anesthetic agents or control agents or those lacking clear cognitive assessment results were excluded. Studies with a primary focus on emergence delirium were also excluded. The focus of the review was on post-operative cognitive function starting 24 hours after the intervention. Lastly, studies not published in English were omitted from this review.

Data Collection

Data collection involved a comprehensive search of multiple databases, including PubMed, Medline, ScienceDirect, Cochrane Library, Europe PMC, ClinicalTrials.gov, and EBSCO Open Dissertations. This was completed on November 17, 2024. The search strategy utilized specific keywords related to each concept of interest. For ketamine, the search included terms such as "Ketamine", "Ketalar", and "Ketamine hydrochloride". For propofol, the keywords included "Propofol" and "Diprivan". The search also targeted the elderly population using terms such as "elderly", "older adults", and "aged". Cognitive function was addressed through keywords such as "cognitive function", "cognitive dysfunction", and "delirium". In databases where Medical Subject Heading (MeSH) terms were an option, these were incorporated. The full details are shown in Table 1.

**Table 1:** Search strategy I/E, inclusion/exclusion; Medline, Medical Literature Analysis and Retrieval System Online; PMC, PubMed Central; EBSCO, Elton B. Stephens Company

Database/register searched	Search strategy (including filters)	Number of papers before (after) I/E criteria application
PubMed/Medline	(("esketamine"[Supplementary Concept] OR "esketamine"[All Fields] OR "ketamine"[All Fields] OR "ketamine"[MeSH Terms] OR "ketamin"[All Fields] OR "ketamine s"[All Fields] OR "ketamines"[All Fields] OR ("esketamine"[Supplementary Concept] OR "esketamine"[All Fields] OR "ketamine"[All Fields] OR "ketamine"[MeSH Terms] OR "ketamin"[All Fields] OR "ketalar"[All Fields] OR "ketamine s"[All Fields] OR "ketamines"[All Fields]) OR "ketamine*"[All Fields] OR "Ketamine hydrochloride"[All Fields] OR ("ketamine"[MeSH Terms] OR "ketamine"[MeSH Terms] OR "ketamine*"[MeSH Terms] OR "ketamine/adverse effects"[MeSH Terms] OR "ketamine/therapeutic use"[MeSH Terms]) OR ("propofol"[MeSH Terms] OR "propofol"[All Fields] OR "propofol s"[All Fields] OR ("propofol"[MeSH Terms] OR "propofol"[All Fields] OR "diprivan"[All Fields] OR "propofol s"[All Fields]) OR "propofol*"[All Fields] OR ("propofol"[MeSH Terms] OR "propofol"[MeSH Terms] OR "propofol*"[MeSH Terms] OR "propofol/adverse effects"[MeSH Terms] OR "propofol/therapeutic use"[MeSH Terms]))) AND ("Aged"[MeSH Terms] OR "Aged"[All Fields] OR "elderly"[All Fields] OR "elderlies"[All Fields] OR "elderly s"[All Fields] OR "elderlys"[All Fields] OR ("elder s"[All Fields] OR "elders"[All Fields] OR "sambucus"[MeSH Terms] OR "sambucus"[All Fields] OR "elder"[All Fields]) OR ("elder s"[All Fields] OR "elders"[All Fields] OR "sambucus"[MeSH Terms] OR "sambucus"[All Fields] OR "elder"[All Fields]) OR "older adults"[All Fields] OR "older adult"[All Fields] OR "adult older"[All Fields] OR "adults older"[All Fields] OR "Aged"[MeSH Terms]) AND ("cognitive function"[All Fields] OR ("delirium"[MeSH Terms] OR "delirium"[All Fields] OR "delirium s"[All Fields] OR "deliriums"[All Fields]) OR "delir*"[All Fields] OR "cognitive dysfunction"[All Fields] OR "cognitive dysfunctions"[All Fields] OR "dysfunction cognitive"[All Fields] OR "dysfunctions cognitive"[All Fields] OR "cognitive impairment"[All Fields] OR "cognitive impairments"[All Fields] OR "impairment cognitive"[All Fields] OR "impairments cognitive"[All Fields] OR "complication postoperative cognitive"[All Fields] OR "Postoperative Cognitive Complication"[All Fields] OR "Postoperative Cognitive Decline"[All Fields] OR "cognitive decline postoperative"[All Fields] OR "Postoperative Cognitive Dysfunction"[All Fields] OR "cognitive dysfunction postoperative"[All Fields] OR "Postoperative Cognitive Disorders"[All Fields] OR "Postoperative Cognitive Disorder"[All Fields] OR ("neurobehavioral manifestations/drug effects"[MeSH Terms] OR "postoperative cognitive complications/chemically induced"[MeSH Terms] OR "postoperative cognitive complications/drug therapy"[MeSH Terms] OR "postoperative cognitive complications/prevention and control"[MeSH Terms] OR "postoperative cognitive complications/surgery"[MeSH Terms] OR "cognitive dysfunction/chemically induced"[MeSH Terms] OR "cognitive dysfunction/classification"[MeSH Terms] OR "cognitive dysfunction/drug therapy"[MeSH Terms] OR "cognitive dysfunction/prevention and control"[MeSH Terms] OR "cognitive dysfunction/surgery"[MeSH Terms] OR "cognition/classification"[MeSH Terms] OR "cognition/drug effects"[MeSH Terms] OR "delirium/classification"[MeSH Terms] OR "delirium/complications"[MeSH Terms] OR "delirium/drug therapy"[MeSH Terms] OR "delirium/prevention and control"[MeSH Terms] OR "delirium/surgery"[MeSH Terms]))). Filters: Free full text, Adaptive Clinical Trial, Clinical Study, Clinical Trial, Controlled Clinical Trial, Equivalence Trial, Observational Study, Pragmatic Clinical Trial, Randomized Controlled Trial, English, Humans	961 (141)
ScienceDirect	((Ketamine OR Ketalar) OR (Propofol OR Diprivan)) AND (“cognitive function” OR “cognitive dysfunction” OR delirium) AND elderly. Filters: English, Research articles, Open access, and Open archive	3,788 (344)
Cochrane Library (CENTRAL)	((Ketamine OR Ketalar OR Ketamine* OR “Ketamine NEXT hydrochloride” OR “Ketamine NEAR Propofol” OR [Ketamine]) OR (Propofol OR Diprivan OR Propofol* OR “Propofol NEAR Ketamine” OR [Propofol])) AND (elderly OR elder OR elders OR “older NEAR adults” OR “older NEAR adult” OR [Aged]) AND (“cognitive NEAR function” OR delirium OR “cognitive NEAR dysfunction” OR “cognitive NEAR dysfunctions” OR “cognitive NEAR impairment” OR “cognitive NEAR impairments” OR “Postoperative NEAR Cognitive Complication” OR “Postoperative NEAR Cognitive Decline” OR “Postoperative NEAR Cognitive Dysfunction” OR “Postoperative NEAR Cognitive Disorders” OR “Postoperative NEAR Cognitive Disorder” OR [Cognition] OR [Delirium] OR [Cognitive Dysfunction] OR [Postoperative Cognitive Complications]) Filters: Trials, English	486 (455)
Europe PMC	((ABSTRACT:"Ketamine" OR ABSTRACT:"Ketalar" OR ABSTRACT:"Propofol" OR ABSTRACT:"Diprivan") AND ABSTRACT:"elderly" AND (ABSTRACT:"cognitive dysfunction" OR ABSTRACT:"cognitive function" OR ABSTRACT:"delirium")). Filters: Full text in Europe PMC, Link to free full text, Research articles	156 (83)
ClinicalTrials.gov	Outcome Measure: (“cognitive function” OR delirium OR delir* OR “cognitive dysfunction” OR “cognitive dysfunctions” OR “dysfunction, cognitive” OR “dysfunctions, cognitive” OR “cognitive impairment” OR “cognitive impairments” OR “impairment, cognitive” OR “impairments, cognitive” OR “Complication, Postoperative Cognitive” OR “Postoperative Cognitive Complication” OR “Postoperative Cognitive Decline” OR “Cognitive Decline, Postoperative” OR “Postoperative Cognitive Dysfunction” OR “Cognitive Dysfunction, Postoperative” OR “Postoperative Cognitive Disorders” OR “Postoperative Cognitive Disorder”) Intervention/treatment: (Ketamine OR Ketalar OR Ketamine* OR “Ketamine hydrochloride”) OR (Propofol OR Diprivan OR Propofol*). Filters: Completed studies | Interventional, Observational studies | Studies with results	422 (29)
EBSCO Open Dissertations	(Ketamine OR Ketalar) OR (Propofol OR Diprivan) AND (“cognitive function” OR delirium OR delir* OR “cognitive dysfunction” OR “cognitive dysfunctions” OR “dysfunction, cognitive” OR “dysfunctions, cognitive” OR “cognitive impairment” OR “cognitive impairments” OR “impairment, cognitive” OR “impairments, cognitive” OR “Complication, Postoperative Cognitive” OR “Postoperative Cognitive Complication” OR “Postoperative Cognitive Decline” OR “Cognitive Decline, Postoperative” OR “Postoperative Cognitive Dysfunction” OR “Cognitive Dysfunction, Postoperative” OR “Postoperative Cognitive Disorders” OR “Postoperative Cognitive Disorder”) AND (elderly OR elder OR elders OR “older adults” OR “older adult” OR “adult, older” OR “adults, older”). Filters: none	215 (215)

The search strategy integrated these concepts to ensure a comprehensive retrieval of relevant literature. Following the initial search, the results underwent screening using the Rayyan application to remove duplicates and assess eligibility based on the predefined criteria. This screening process included a general review of titles and abstracts, followed by a full-text assessment for studies that met the inclusion criteria.

Data Extraction and Quality Assessment

Data extraction was conducted using a standardized form to collect key information from the included studies, such as study design, population characteristics, intervention details, cognitive assessment tools used, and outcomes measured. To ensure the reliability and validity of the findings, the methodological quality of the included studies was assessed using appropriate tools. The Cochrane Risk-of-Bias-2 (RoB2) Tool was employed for RCTs [22], while the Newcastle-Ottawa Scale (NOS) was utilized for observational studies [23].

Data Synthesis

The data synthesis was performed narratively, summarizing the findings from the included studies to provide an overview of how ketamine and propofol influenced cognitive function in the elderly population. Results were organized into tables to facilitate comparison across studies. This approach allowed for a comprehensive understanding of existing evidence and highlighted any gaps in the current literature. A meta-analysis was not performed due to a high heterogeneity of the included studies. This was due to variability in anesthetic protocols, dosages, and tools used to measure outcomes, which collectively limit direct comparability.

Results

**Figure 1:**
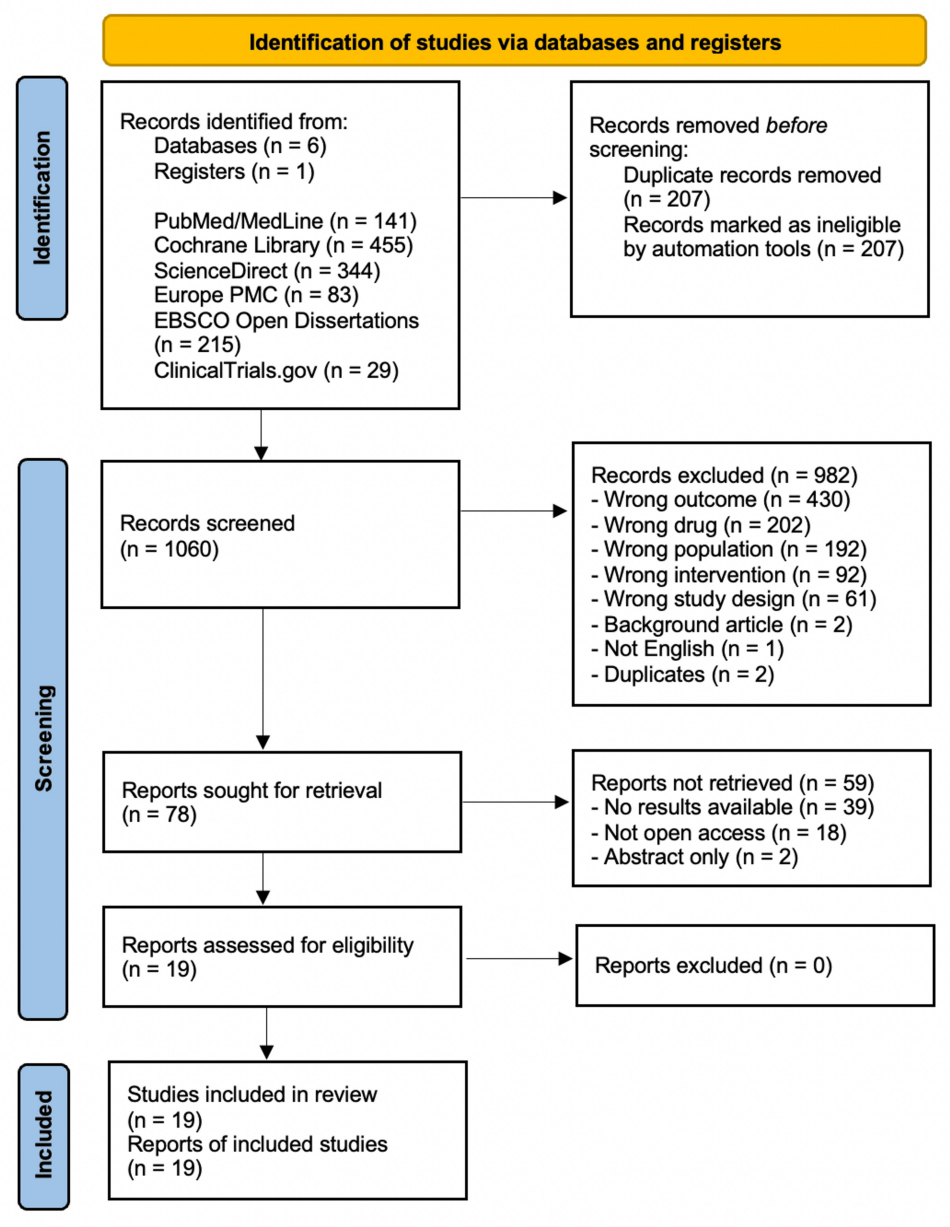
PRISMA flow diagram PRISMA, Preferred Reporting Items for Systematic Review and Meta-Analysis; Medline, Medical Literature Analysis and Retrieval System Online; PMC, PubMed Central; EBSCO, Elton B. Stephens Company

Following the completion of the search strategy, a total of 1,267 studies were identified across six databases and one register. After removing duplicates using the Rayyan application and applying the predefined inclusion and exclusion criteria [24], 78 studies were sought for full-text retrieval. Ultimately, 19 studies met the eligibility criteria and were included in this systematic review, as illustrated in the PRISMA flow diagram (Figure 1).

Risk-of-Bias Assessment

The risk of bias for the included studies was assessed to ensure the reliability of the findings. Table 2 summarizes the risk of bias for 17 RCTs evaluated using the Cochrane RoB2 Tool [22]. Among these, 12 studies demonstrated a low risk across all domains, two studies exhibited “some concerns” in domain 3, and three studies showed “some concerns” in both domains 2 and 3. Domain 2 concerns bias that stems from changes to intended interventions, and domain 3 concerns bias originating from outcome data that is missing [22]. None of the studies were classified as high risk, thereby maintaining their inclusion in the review.

**Table 2:** Bias assessment of RCTs Note: Cochrane RoB2 Tool assesses five domains: domain 1 (bias arising from the randomization process), Domain 2 (bias due to deviations from intended interventions), domain 3 (bias due to missing outcome data), domain 4 (bias in measurement of the outcome), and domain 5 (bias in selection of the reported result(s)) [22]. Each domain is scored either a) low risk (✓), b) some concerns (±), or c) high risk (X) [22]. RCT, randomized clinical trial

Study	Domain 1	Domain 2	Domain 3	Domain 4	Domain 5	Overall
Abd Ellatif et al., 2024 [13]	✓	✓	✓	✓	✓	✓
Zhang et al., 2018 [15]	✓	✓	✓	✓	✓	✓
Tian et al., 2017 [16]	✓	✓	✓	✓	✓	✓
Shin et al., 2023 [17]	✓	✓	✓	✓	✓	✓
Ghazaly et al., 2023 [18]	✓	✓	✓	✓	✓	✓
Wittwer et al., 2023 [19]	✓	±	±	✓	✓	±
Siripoonyothai and Sindhvananda, 2021 [20]	✓	✓	✓	✓	✓	✓
Rasmussen et al., 2006 [25]	✓	±	±	✓	✓	±
Zhang et al., 2022 [26]	✓	✓	✓	✓	✓	✓
Zhi and Li, 2023 [27]	✓	±	±	✓	✓	±
Li et al., 2023 [28]	✓	✓	±	✓	✓	±
Sieber et al., 2018 [29]	✓	✓	✓	✓	✓	✓
Zhu et al., 2023 [30]	✓	✓	✓	✓	✓	✓
Mei et al., 2020 [31]	✓	✓	✓	✓	✓	✓
Ding et al., 2021 [32]	✓	✓	✓	✓	✓	✓
Royse et al., 2011 [33]	✓	✓	✓	✓	✓	✓
Verdonk et al., 2024 [34]	✓	✓	±	✓	✓	±

Table 3 presents the risk-of-bias assessment for the two cohort studies included in this review, evaluated using the NOS. The Agency for Healthcare Research and Quality states that a score of 7 or above is a good quality resource; only good quality articles were included [23]. One of the studies received a maximum score of 9/9, indicating good quality, while the other study scored 8/9, also reflecting good quality.

**Table 3:** Quality assessment for cohort studies using NOS Note: NOS permits four stars (*) for selection, two for comparability, and three for outcome. Total scores range from 0 to 9. AHRQ quality thresholds state 7 or above as “good quality” [23]. NOS, Newcastle Ottawa Scale; AHRQ, Agency for Healthcare Research and Quality

Authors	Study design	Selection	Comparability	Outcome	Total score	Overall quality
Tekletsadik et al., 2024 [12]	Multicenter prospective cohort	****	**	***	9/9	Good quality
Yang et al., 2023 [35]	Retrospective cohort	****	*	***	8/9	Good quality

Summary of Included Studies

The systematic review includes a total of 19 studies, comprising 17 RCTs and 2 cohort studies. These studies collectively assessed the effects of ketamine and propofol on cognitive function in elderly patients undergoing anesthesia. The studies were conducted globally, with nine studies originating from China, followed by two from the USA, two from Egypt, and one each from Denmark, Thailand, Australia, France, Korea, and Ethiopia. Sample sizes varied widely, ranging from 41 to 748 participants.

The studies exhibited considerable variability regarding which anesthetic agents were investigated. Three studies directly compared ketamine and propofol [12,19,20], while 12 focused exclusively on propofol [15-17,25-33], and three on ketamine alone [18,34,35]. Additionally, studies examined other anesthetic agents, including xenon [25], remimazolam [26], combinations of etomidate [27,35], dexmedetomidine [13,17,18,30], sevoflurane [15,16,31,32], and desflurane (in combination with sevoflurane) [33]. Only three studies utilized a placebo (0.9% normal saline) [13,18,34]. Two studies specifically investigated ketofol [12,13].

Most studies specified a surgical subspecialty, except for two studies, encompassing various surgical specialties [18,27]. Seven studies concentrated on orthopedic surgery [17,25,26,29-31,34], four on cardiac surgery (one specifically on cardiopulmonary bypass) [19,20,33,35], three on gastrointestinal surgery (one specifically on colorectal/rectal surgery) [13,28,32], one on thoracic surgery (specifically lung cancer resection) [16], and one on major cancer surgery (excluding neurosurgery) [15]. Six studies did not specify the dosages used [12,15,17,29,31,33].

The most common methods employed to assess POCD and POD included the Confusion Assessment Method (CAM), MMSE, MoCA, and various neuropsychological tests.

Among the studies investigating propofol, seven focused on maintenance anesthesia, four on induction, two on sedation, one on cardiopulmonary bypass, and one on both induction and maintenance [12,15-17,19,20,25-33]. Two studies primarily examined lighter versus heavier sedation [29,30], while another assessed the impact of varying propofol infusion rates during induction [28]. Propofol doses ranged from 0.5 to 2 mg/kg for induction, whereas maintenance dosages ranged from 4 to 8 mg/kg/h [16,19,25-27]. Some studies reported a target plasma concentration of 1.5 to 4 μg/mL instead of a specific dosage range [32,33].

The studies focusing on ketamine explored its use as boluses before or after induction, during induction, as an anesthetic adjunct, and specifically during cardiopulmonary bypass [12,18-20,34,35]. Ketamine dosages ranged from 1 to 2 mg/kg for induction [19], with bolus doses of 0.5 or 1.0 mg/kg [18,34].

One ketofol study concentrated on induction, while the other assessed ketofol as an anesthetic adjunct [12,13]. The dosage for ketofol as an adjunct was 0.3-0.4 mg/kg/h [13]. Further details can be found in Table 4, which summarizes the included studies.

**Table 4:** Summary of included studies RCT, randomized controlled trials; POCD, postoperative cognitive dysfunction; MMSE, Mini-Mental State Examination; HVLT-R, Hopkins Verbal Learning Test-Revised; COWAT, Controlled Oral Word Association Test; CPB, cardiopulmonary bypass; CAM-ICU, Confusion Assessment Method-Intensive Care Unit; MoCA, Montreal Cognitive Assessment; POD, postoperative delirium; MAC, minimum alveolar concentration; OR, odds ratio; GA, general anesthesia; RR, relative risk; IM, intramuscular; BIS, bispectral index; COR, crude odd ratio; AOR, adjusted odds ratio

Study	Study design	Country	Number of participants	Aim of study	Intervention	Reported outcomes	Additional key findings
Tekletsadik et al., 2024 [12]	Multi-center prospective cohort	Ethiopia	220	POD among elderly elective orthopedic patients in Addis Ababa Ethiopia	Ketamine (induction, no dosage), propofol (induction, no dosage), ketofol (induction, no dosage)	CAM. Ketamine POD: 1/14 (COR: 1.24 [95% CI: 1.13-4.032], AOR: 1.32 [95% CI: 1.11-3.87], P=0.003). Propofol POD: 6/26 (COR: 0.115 95% CI: [0.11-1.76], AOR: 0.14 [95% CI: 0.12-2.55], P=0.049). Ketofol POD: 6/15 (COR: 0.26 [95% CI: 0.19-3.2], AOR: 0.22 [95% CI: 0.15-1.98], P=0.061). All compared to thiopentone POD: 0/1.	70 (31.8%) underwent upper extremity surgery, 150 (68.1%) underwent lower extremity surgery. 164 (75.6%) received spinal anesthesia or peripheral nerve block, 14 (6.4%) received GA with a face mask, and 42 (19.1%) received GA with endotracheal intubation. 14 (25%) were induced by ketamine, 26 (46.4%) with propofol, 15 (26.8%) with ketofol, and 1 (1.8%) with thiopentone. Small sample sizes.
Abd Ellatif et al., 2024 [13]	RCT	Egypt	120	Ketofol vs. dexmedetomidine for preventing POD in elderly patients undergoing intestinal obstruction surgeries	Ketofol (0.3-0.4 mg/kg/h, i.e., propofol 0.3-0.4 mg/kg/h and ketamine 0.125 mg/kg/h, given during surgery and 2 hours postoperatively) vs. dexmedetomidine (0.2 µg/kg/h) vs. placebo (21 mL 0.9% saline, 0.3-0.4 mg/kg/h)	CAM-ICU. Incidence of POD significantly higher in the placebo group than in the ketofol group (P<0.05). No statistical significance between other groups.	Induction: fentanyl (2 µg/kg), propofol (1.5-2 mg/kg), and rocuronium (1 mg/kg). Maintenance: 1-1.5 MAC isoflurane and rocuronium (0.2 mg/kg every 30 min). Fentanyl (0.5 µg/kg) as needed to maintain anesthetic depth. Sugammadex (2-4 mg/kg) at the end of surgery. Postoperative analgesia was paracetamol (15 mg/kg 4x/day) and ketorolac (0.5 mg/kg 3x/day).
Zhang et al., 2018 [15]	RCT	China	392	Propofol compared with sevoflurane GA is associated with decreased delayed neurocognitive recovery in older adults	Sevoflurane (target-controlled, for maintenance, no dosage) vs. propofol (target-controlled infusion for maintenance, no dosage)	Neuropsychological tests. Propofol POCD 14.8% (28/189) vs. sevoflurane POCD 23.2% (44/190), OR 0.577 (95% CI: 0.342-0.975), P=0.038. Per-protocol analysis: propofol POCD 14.3% (26/182) vs. sevoflurane POCD 24.6% (42/171), OR 0.512 (95% CI: 0.298-0.880), P=0.014. Study included a control group but did not compare individual anesthetic agents.	Induction: midazolam, remifentanil, propofol, rocuronium, or cisatracurium and/or sufentanil. Postoperative analgesia: morphine (0.5 mg/mL) or sufentanil (1-2 μg/mL). 1 surgery was cancelled in the propofol group and 4 in the sevoflurane group. 8 deviated from protocol in the propofol group and 19 in the sevoflurane group. 6 lost to follow-up in the propofol group and 2 in the sevoflurane group. Major cancer surgery excluding neurosurgery.
Tian et al., 2017 [16]	RCT	China	62	Effects of propofol or sevoflurane anesthesia on the perioperative inflammatory response, pulmonary function, and cognitive function in patients receiving lung cancer resection	Sevoflurane (induction with 8% and maintenance with 2%) vs. propofol (induction with 1 mg/kg and maintenance with 6 mg/kg/h)	MMSE for POCD. The propofol group had significantly higher scores at 24 hours than the sevoflurane group (P<0.05).	Prior to induction: midazolam (0.1 mg/kg) and fentanyl (3 μg/kg).
Shin et al., 2023 [17]	RCT	Korea	748	POD after dexmedetomidine vs. propofol sedation in healthy older adults undergoing orthopedic lower limb surgery with spinal anesthesia	Dexmedetomidine (loading dose of 1 μg/kg and then 0.1-0.5 µg/kg/h; stopped 30 min earlier than propofol) vs. propofol (continuously infused via a target-controlled infusion device, adjusting the effect-site concentration within 1-2 μg/mL)	CAM. Dexmedetomidine POD 3.0% vs. propofol POD 6.6%, OR 0.42 (95% CI: 95% CI: 0.201-0.86, P=0.036).	Spinal anesthesia with 0.5% hyperbaric bupivacaine (2-3 mL) and fentanyl (10-20 μg). After allocations, 8 in each group withdrew consent or had their operation cancelled. 25 in the propofol group and 24 in the dexmedetomidine group deviated from protocol and were excluded due to not receiving sedation, having sedation stopped or changing to GA.
Ghazaly et al., 2023 [18]	RCT	Egypt	60	A pre-anesthetic bolus of ketamine vs. dexmedetomidine for prevention of POD in elderly patients undergoing emergency surgery	Ketamine (1 mg/kg 10 min before induction) vs. dexmedetomidine (1 µg/kg) vs. placebo (0.9% saline)	Tool to measure not given. Placebo POD 15 (75%) vs. dexmedetomidine POD 1 (5%) vs. ketamine POD 2 (10%), P<0.001. Ketamine POD OR 3.012 (95% CI: 1.185-9.681), P=0.013. Placebo POCD 7 (35%) vs. dexmedetomidine POCD 0 (0%) vs. ketamine POCD 2 (10%), P=0.006. Ketamine POCD OR 4.501 (95% CI: 1.161-8.817), P=0.006.	Induction: fentanyl (1 µg/kg), propofol (1-2 mg/kg), and rocuronium (1 mg/kg). Maintenance: sevoflurane. Given neostigmine and atropine postoperatively. Only emergency gastrointestinal, orthopedic, vascular, obstetric, urologic, or plastic surgery.
Wittwer et al., 2023 [19]	RCT	USA	52	Impact of ketamine vs. propofol for anesthetic induction on cognitive dysfunction, delirium, and acute kidney injury following cardiac surgery in elderly, high-risk patients	Ketamine (1-2 mg/kg for induction) vs. propofol (0.5-1 mg/kg for induction)	Trail Making Test A and B, MMSE, HVLT-R, Digit Span, COWAT, the Stroop Color and Word Test. Ketamine POCD (64%) vs. Propofol POCD (43%), P=0.23.	Additional medications as needed for intubation. Inhalation anesthetics, muscle relaxants, and opioids permitted during induction. Maintenance: isoflurane. Dexmedetomidine (0.5–1.5 μg/kg/h) after surgery. 2 in the ketamine group and 1 in the propofol group did not receive their intervention.
Siripoonyothai and Sindhvananda, 2021 [20]	RCT	Thailand	75	Comparison of POD within 24 hours between ketamine and propofol infusion during CPB machine	Ketamine (1 mg/kg/h during CPB) vs. propofol (1.5-6 mg/kg/h during CPB)	Thai-CAM-ICU. Ketamine POD: 10 (31.25%). Propofol POD: 18 (56.25%). P=0.04. After multivariate logistic regression analysis, this was not significant.	Induction: etomidate (0.2-0.3 mg/kg), fentanyl (1-2 μg/kg), and cisatracurium (0.15 mg/kg). Maintenance: cisatracurium (1.5 μg/kg/min) and fentanyl (0.5-1 μg/kg/h). Sevoflurane (1-2%) and midazolam (0.02-0.05 mg/kg) permitted to maintain anesthetic depth. Postoperative analgesia with fentanyl (0.5-1.5 μg/kg/h). 5 lost to follow-up in the ketamine group and 6 in the propofol group.
Rasmussen et al., 2006 [25]	RCT	Denmark	41	Comparison of xenon with propofol for supplementary GA for knee replacement	Xenon (50-70% for maintenance) vs. propofol (3-5 mg/kg/h for maintenance)	Neuropsychological testing. POCD at discharge 7/20 (35.0%, 15-59%) vs. 6/16 (37.5%, 15-65%) for xenon and propofol groups, respectively (P=0.88). At 3 months, 3/18 (16.7%, 4-41%) vs. 2/16 (12.5%, 2-38%) for the xenon and propofol groups, respectively (P=0.77).	Midazolam (1-5 mg) before regional anesthesia. Spinal anesthesia with bupivacaine (15 mg) and postoperative intrathecal morphine (0.1 mg) or epidural catheter (sufentanil). Induction: propofol (1-2 mg/kg). Propofol or alfentanil given if upper body movement detected. Postoperative analgesia included oral acetaminophen and oral/IV morphine. 39 received medication, 3 lost to follow-up, leaving 20 in the xenon group and 16 in the propofol group. Small sample size, not powered to show difference in POCD.
Zhang et al., 2022 [26]	RCT	China	60	Application effects of remimazolam and propofol on elderly patients undergoing hip replacement	Remimazolam (0.2-0.4 mg/kg for induction, 0.3-0.5 mg/kg/h for maintenance) vs. propofol (1.5-2 mg/kg for induction, 4-8 mg/kg/h for maintenance)	MMSE for POCD propofol. Before induction: 26.99 ± 2.41. At 1 day: 16.74 ± 1.76. At 3 days: 19.37 ± 2.08. At 7 days: 25.77 ± 2.51. Remimazolam. Before induction: 27.31 ± 2.36. At 1 day: 20.64 ± 1.99. At 3 days: 22.64 ± 2.31. At 7 days: 26.99 ± 2.49. Both propofol and remimazolam MMSE scores, on day 1 and 3, were significantly lower than before induction (P<0.05). On day 1 and 3, propofol MMSE scores were significantly lower than remimazolam (P<0.05).	Iliac fascia block with 40 mL 0.25% ropivacaine. Induction: sufentanil (0.4 μg/kg) and cisatracurium (0.15 mg/kg). Maintenance: remifentanil (0.1–0.25 μg/kg/min). Both groups received flumazenil (0.3 mg) after surgery ended.
Zhi and Li, 2023 [27]	RCT	China	140	Effects of total intravenous anesthesia with etomidate and propofol on POD	Propofol-etomidate (0.3 mg/kg) vs. propofol (1-2 mg/kg for induction)	MMSE/MoCA for POCD. In both groups, scores were significantly reduced after the operation (at 24 hours and 72 hours) compared to before. Compared with propofol, the combination including etomidate reduced POCD at 24 hours and at 72 hours (P<0.05).	Etomidate group was in combination with the same dose of propofol. Induction: remifentanil (0.05-1 μg/kg), midazolam (0.04 mg/kg), propofol (1-2 mg/kg), and cisatracurium (0.10-0.15 mg/kg). Maintenance: not specified. 7 in the primary group removed and 10 in the control group removed during the study. Included patients undergoing a variety of different specialty surgeries.
Li et al., 2023 [28]	RCT	China	180	Effects of different injection rates of propofol on postoperative cognition in elderly patients undergoing laparoscopic inguinal hernia repair	Group 1: propofol (30 mg/kg/h) vs. group 2: propofol (100 mg/kg/h) vs. group 3: propofol (300 mg/kg/h)	MMSE, MoCA. Group 1 POCD 8.6% vs. group 2 POCD 11.9% vs. group 3 POCD 16.9% at 24 hours, P=0.389. Group 1 POCD 3.4% vs. group 2 POCD 5.1% vs. group 3 POCD 8.5% at 7 days, P=0.493.	Induction: group + sufentanil (0.4 µg/kg) and rocuronium (0.6 mL/kg). Maintenance: propofol (4-8 mL/kg/h) and remifentanil (0.1-0.3 μg/kg/min). At the end of surgery: ondansetron 4 mg and flurbiprofen axetil 100 mg. Postoperative analgesia was with flurbiprofen axetil (50 mg). 2 patients lost to follow-up in group 1, 1 in group 2, and 1 in group 3. Men only.
Sieber et al., 2018 [29]	RCT	USA	200	Effect of depth of sedation in older patients undergoing hip fracture repair on POD	Propofol (lighter vs. heavier sedation, decided by multiple anesthetic depth measurements)	CAM, Delirium-Rating Scale Revised-98, Digit Span. Incidence of POD in postoperative days 1-5 was 34% in the lighter group and 39% in the heavier group (P=0.46).	Diagnosis of delirium via multidisciplinary panel including medical records, family/nursing staff interviews, and measurements mentioned to the left. All patients underwent spinal anesthesia. 4 in group 1 and 3 in group 2 lost to follow-up.
Zhu et al., 2023 [30]	RCT	China	226	Different sedation strategies in older patients receiving spinal anesthesia for hip surgery on POD	Propofol (as sedative at 0.5-3.0 mg/kg/h, sedation level either lighter (<2) or heavier (>3) using Modified Observer’s Assessment of Alertness and Sedation score) vs. dexmedetomidine (loading dose of 0.3 μg/kg, then 0.2-0.7 μg/kg/h, sedation level either lighter or heavier as above)	CAM. Dexmedetomidine POD (11.9%) vs. propofol POD (23.6%), RR 0.51 (95% CI: 0.274-0.929), P=0.024. Overall incidence of POD in propofol lighter (14.5%) vs. propofol heavier (32.7%) groups, RR 2.25 (95% CI: 1.069-4.736), P=0.025. Hypoactive POD in propofol lighter (1.8%) vs. propofol heavier (20.0%) groups, RR 11.0 (95% CI: 1.470-82.319), P=0.002. Severe POD in propofol lighter (5.5%) vs. propofol heavier (23.6%) groups, RR 4.33 (95% CI: 1.307-14.365), P=0.013. No significant differences between dexmedetomidine lighter and heavier groups.	Spinal anesthesia with 0.25% bupivacaine (2 mL). Only assessed cognitive function during the first 72 hours. 7 patients excluded after randomization.
Mei et al., 2020 [31]	RCT	China	240	The effects of propofol and sevoflurane on POD in older patients	Sevoflurane (1-4% for maintenance) vs. Propofol (using target-controlled infusion (629.8 ±255.0 mg) for maintenance)	CAM. Incidence of POD propofol 33.0% (35/106) vs. sevoflurane 23.3% (24/103), P=0.119. POD severity propofol 2.5 ±1.2 vs. sevoflurane 2.3 ±1.2, P=0.364. Days of POD propofol 0.5 ±0.8 vs. sevoflurane 0.3 ±0.5, P=0.049.	Induction: propofol (2 mg/kg), sufentanil (0.5-1 μg/kg), and cisatracurium (0.5 mg/kg). Other drugs: preoperative midazolam (1–2 mg), methylprednisolone (40-80 mg) to prevent allergic reaction from bone cement and atropine (0.25-1 mg) for airway secretions. 4 in the sevoflurane group and 8 in the propofol group did not receive allocated anesthetic. 11 in the sevoflurane group and 8 in the propofol group lost to follow-up. Only elective total hip/knee replacements.
Ding et al., 2021 [32]	RCT	China	130	Effect of propofol-based total intravenous anesthesia on postoperative cognitive function and sleep quality in elderly patients	Sevoflurane (1.2%-2.3% for maintenance) vs. propofol (target plasma concentration of 2-4 µg/mL during maintenance)	MMSE for POCD. On postoperative days 1, 3, 7, and 15, propofol group scores were higher than the sevoflurane group scores, P<0.001).	Induction: cisatracurium (0.1- 0.15 mg/kg), sufentanil (0.2- 0.4 g/kg), and etomidate (0.2-0.3 mg/kg). Sufentanil (0.2-0.3 µg/kg) and cisatracurium (0.04-0.06 mg/kg) as needed to maintain anesthetic depth. Postoperative analgesia: IM parecoxib sodium 40 mg 2x/day for 2 days. Only rectal or colon surgery.
Royse et al., 2011 [33]	RCT	Australia	182	The inﬂuence of propofol or desﬂurane on POD in patients undergoing coronary artery bypass surgery	Desflurane group underwent induction with sevoflurane, then maintenance with desflurane, titrated as needed, to maintain BIS 40-60) vs. propofol (for induction a target concentration infusion of 1.5-3 μg/mL, then maintenance, titrated as needed, to maintain BIS 40-60)	Neuropsychological testing for POCD/CAM for POD. At hospital discharge: propofol POCD 67.5% (56/84) vs. desflurane POCD 49.4% (41/83), P=0.018. At 3 months: propofol POCD 11.2% (10/87) vs. desflurane POCD 10.0% (9/90), P=0.748. POD during hospital stay: propofol 16 (18%) vs. desflurane 18 (19.8%), P=0.757.	Induction (both groups): fentanyl (2-5 μg/kg) and midazolam (0.025-0.05 mg/kg). Maintenance (both groups): fentanyl (1.5 μg/kg/h) and midazolam (0.025-0.05 mg/kg/h). Postoperatively (both groups): propofol (50-150 mg/h) until ready for extubation. 2 in the propofol group and 1 in the desflurane group lost to follow-up. A further 2 in the propofol group were excluded due to not receiving trial anesthetic.
Verdonk et al., 2024 [34]	RCT	France	301	Preoperative ketamine administration for prevention of postoperative neurocognitive disorders after major orthopedic surgery in elderly patients	Ketamine (0.5 mg/kg bolus after induction) vs. placebo (equal volume 0.9% saline)	MoCA, Trail Making Test A and B for POCD / CAM for POD. Day 7: ketamine POCD 50 (38.8%) vs. placebo POCD 54 (40.9%), OR 0.92 (95% CI: 0.56-1.51), P=0.73. On day 90: ketamine POCD 26 (20.8%) vs. placebo POCD 23 (20%), OR 1.05 (95% CI: 0.56-1.97), P=0.884. Day 7: ketamine POD 6 (4.14%) vs. placebo POD 9 (6.34%), OR 0.80 (95% CI: 0.26-2.47), P=0.698. Per-protocol analysis findings were also insignificant.	Included patients with preoperative cognitive impairment: no significant differences on examining subgroups. Further anesthesia at the discretion of the anesthetic team. 3 withdrew consent in the ketamine group, 6 in the placebo group. 20 had no available primary outcome data on day 7 (POCD) in the ketamine group, 11 in the placebo group. 4 excluded in the ketamine group from per-protocol analysis, 22 from the placebo group.
Yang et al., 2023 [35]	Retrospective cohort	China	100	Effect of combined etomidate-ketamine anesthesia on perioperative electrocardiogram and POD of elderly patients with rheumatic heart valve disease undergoing heart valve replacement	Ketamine-etomidate (0.3 mg/kg) vs. ketamine (5 μg/kg/min until end of the surgery)	MoCA. Ketamine POCD 20.0% (10/50) vs. ketamine-etomidate POCD 6.0% (3/50), P<0.05.	The ketamine-etomidate cohort received the same dose of ketamine as the ketamine-only cohort. Preoperatively: morphine, IM scopolamine (0.3 mg), and sufentanil (0.1 mg). Induction: midazolam (0.05-0.08 mg/kg), fentanyl (10-15 μg/kg), pipecuronium (0.08-0.10 mg/kg), and low-dose ketamine (0.5 mg/kg). Maintenance: midazolam (0.03-0.06 mg/kg), fentanyl (5-10 μg/kg), and pipecuronium (0.05-0.08 mg/kg).

Discussion

This systematic review assesses the impact of ketamine and propofol on cognitive function in elderly patients undergoing anesthesia. While findings indicate anesthetic agents can influence cognitive outcomes, significant variability exists based on dosage, protocol, administration methods, and patient demographics. In particular, studies that directly compared ketamine and propofol revealed nuanced differences in the incidence of POCD and POD, underscoring the need for tailored anesthesia protocols in this vulnerable population.

Comparative Analysis of Ketamine and Propofol on Cognitive Function in Elderly Patients

A comparative analysis of three studies conducted in the USA, Thailand, and Ethiopia offers valuable insights into the effects of ketamine and propofol on cognitive function in elderly patients. Each study examined distinct surgical contexts: one focused on cardiac surgery, another on cardiopulmonary bypass, and the third on elective orthopedic surgery [12,19,20]. Notably, while two of these studies utilized the CAM to measure POD [12,20], the third employed a diverse array of cognitive tests to assess both POCD and POD [19].

Wittwer et al. specifically investigated the use of ketamine and propofol during induction [19]. Their findings indicated an incidence of POCD of 64% for ketamine compared to 43% for propofol; however, this difference was not statistically significant (P = 0.23) [19]. It is essential to note that dexmedetomidine was administered postoperatively, which may complicate the interpretation of these results [19]. In contrast, Tekletsadik et al. conducted a multicenter prospective cohort study where the focus was not solely on the anesthetic drugs administered [12]. In this study, 40 patients received either ketamine or propofol out of the 220 randomized participants [12]. The incidence of POD for ketamine was one out of 14 patients, yielding a crude odds ratio (COR) of 1.24 (95% CI: 1.13-4.032) and an adjusted odds ratio (AOR) of 1.32 (95% CI: 1.11-3.87), with a P-value of 0.003 [12]. This result was statistically significant when compared to the thiopentone-induced group, which included one patient who did not experience POD [12]. Conversely, the incidence of POD for propofol was six out of 26 patients, resulting in a COR of 0.115 (95% CI: 0.11-1.76) and an AOR of 0.14 (95% CI: 0.12-2.55), with a P-value of 0.049 [12]. While this P-value indicates statistical significance, the confidence intervals for both the COR and AOR suggest that the results may not be clinically significant.

Furthermore, Siripoonyothai and Sindhvananda concluded that ketamine attenuated POD compared to propofol, with 31.25% of patients experiencing delirium in the ketamine group (10 total) versus 56.25% in the propofol group (18 total), yielding a P-value of 0.04 [20]. However, while this finding was statistically significant, it lost significance upon multivariate logistic regression analysis [20]. All three studies included various other anesthetic agents for induction and maintenance, which could have further influenced the results regarding the effects of ketamine and propofol on cognitive function in the elderly [12,19,20]. The number of participants per anesthetic drug group varied, with the lowest being 14 and the highest being 38, although after accounting for follow-up losses, the range was between 14 and 32 participants per group [12,19,20].

Evaluating Propofol’s Impact on Cognitive Function in Elderly Surgical Patients Relative to Other Anesthetics

All 12 studies evaluating the effect of propofol on the cognitive function of elderly surgical patients were RCTs [15-17,25-33]. The majority of these studies were conducted in China (eight studies), with additional research originating from Denmark, the USA, Korea, and Australia [15-17,25-33]. The surgical contexts varied significantly, encompassing six studies focused on orthopedic procedures, two on gastrointestinal surgeries, and one each on cardiac, thoracic, major cancer surgeries, and other specialties [15-17,25-33]. Cognitive assessments predominantly utilized methods such as the MMSE, CAM, MoCA, and various neuropsychological tests, with some studies incorporating additional cognitive evaluations.

Among the studies, six Chinese RCTs and one Danish RCT specifically investigated POCD associated with propofol [15,16,25-28,32]. Notably, Li et al. explored the effects of different propofol infusion rates, categorizing participants into three distinct groups consisting solely of male subjects [28]. At 24 hours post-surgery, the rates of POCD were 8.6% for group 1 (propofol at 30 mg/kg/h), 11.9% for group 2 (100 mg/kg/h), and 16.9% for group 3 (300 mg/kg/h), with a P-value of 0.389 indicating no significant differences between groups [28]. On day 7, POCD rates were 3.4%, 5.1%, and 8.5% for groups 1, 2, and 3, respectively, with a P-value of 0.493, further supporting the lack of significant impact on POCD across varying propofol dosages [28].

Zhi and Li conducted a comparative study examining propofol in conjunction with etomidate, administered as an anesthetic adjunct during induction [27]. Both groups exhibited significant reductions in MMSE and MoCA scores postoperatively at both 24 and 72 hours compared to preoperative assessments [27]. However, the combination therapy notably reduced the incidence of POCD at both intervals, achieving statistical significance (P < 0.05) [27]. This study's generalizability was enhanced by its inclusion of patients from various surgical specialties [27].

Zhang et al. provided a comparative analysis between propofol and remimazolam, utilizing both agents for induction and maintenance [26]. Pre-induction MMSE scores were significantly higher for both groups (propofol: 26.99 ± 2.41; remimazolam: 27.31 ± 2.36), indicating baseline cognitive function differences. Postoperatively, scores on days 1 and 3 revealed significant declines in both groups, with propofol scores being lower than those for remimazolam (P < 0.05) [26]. On day 7, the scores for propofol and remimazolam were 25.77 ± 2.51 and 26.99 ± 2.49, respectively, with no significant differences noted [26]. Both groups received flumazenil post-surgery, which likely influenced the outcomes, particularly in the remimazolam cohort [26].

Rasmussen et al. explored the effects of xenon, an inhaled anesthetic, in comparison to propofol [25]. They reported similar rates of POCD at discharge (37.5% for propofol vs. 35.0% for xenon, P = 0.88) and at three months (12.5% for propofol vs. 16.7% for xenon, P = 0.77) [25]. However, the study's design permitted additional propofol administration if upper body movement was detected, which could have confounded the results [25]. Furthermore, the small sample size limited the study's power to detect differences in POCD between the two anesthetics [25].

The remaining three studies comparing propofol to sevoflurane, another inhaled anesthetic, reported varying findings. Zhang et al. found that the incidence of POCD was 14.8% for propofol and 23.2% for sevoflurane, yielding an odds ratio (OR) of 0.577 (95% CI: 0.342-0.975, P = 0.038) [15]. These results remained significant in per-protocol analysis [15]. Ding et al. observed significantly higher MMSE scores for propofol across multiple postoperative days compared to sevoflurane (P < 0.001) [32]. Tian et al. corroborated these findings, noting that propofol scores were significantly higher at 24 hours post-surgery (P < 0.05) [16].

A study in Australia by Royse et al. investigated the cognitive effects of desflurane, a similar inhaled anesthetic, compared to propofol [33]. Although the desflurane group initially underwent induction with sevoflurane, they also received propofol postoperatively [33]. The study reported a higher incidence of POCD in the propofol group at discharge (67.5% vs. 49.4% for desflurane, P = 0.018), although this difference was not significant at the three-month follow-up (11.2% for propofol vs. 10.0% for desflurane, P = 0.748) [33]. Additionally, the incidence of POD was similar between groups, with propofol showing 18.0% incidence compared to 19.8% for desflurane (P = 0.757) [33].

Two Chinese RCTs, one American, and one Korean RCT specifically examined POD [17,29-31]. Two of these studies focused on the effects of varying sedation levels with propofol, with one also incorporating dexmedetomidine as a comparative agent [29,30]. Sieber et al. reported a 34% incidence of POD in the lightly sedated group and 39% in the heavily sedated group (P = 0.46) [29], while Zhu et al. found contrasting results, with lighter sedation leading to a 14.5% incidence of POD compared to 32.7% with heavier sedation (relative risk [RR], 2.25 [95% CI: 1.069-4.736], P = 0.025) [30]. Notably, hypoactive POD was significantly more prevalent in the heavily sedated group (20.0% vs. 1.8%, RR 11.0 [95% CI: 1.470-82.319], P = 0.002), as was severe POD (23.6% vs. 5.5%, RR 4.33 [95% CI: 1.307-14.365], P = 0.013) [30]. The Korean RCT, which included 748 participants, echoed these findings, revealing a significantly lower incidence of POD with dexmedetomidine (3.0%) compared to propofol (6.6%, OR 0.42 [95% CI: 0.201-0.86], P = 0.036) [17]. Lastly, Mei et al. assessed the effects of propofol and sevoflurane on POD, finding no significant differences between groups in terms of POD incidence (33.0% for propofol vs. 23.3% for sevoflurane, P = 0.119) [31]. However, propofol patients experienced a longer average duration of POD (0.5 ± 0.8 days) compared to sevoflurane patients (0.3 ± 0.5 days, P = 0.049) [31].

Evaluating Ketamine’s Impact on Cognitive Function in Elderly Surgical Patients Relative to Other Anesthetics

Three studies conducted in China, Egypt, and France investigated the effects of ketamine on cognitive function in elderly patients. Each study focused on different surgical contexts: cardiac surgery (specifically heart valve replacement in rheumatic disease), emergency surgeries, and orthopedic surgery [18,34,35]. Additionally, one study utilized the MoCA to measure POCD [35], while another employed both the MoCA and Trail Making Test A and B for POCD, along with the CAM for POD [34]. The third study provided results for both POCD and POD but did not specify which tools were used to measure these outcomes [18].

Ghazaly et al. and Verdonk et al., both RCTs, investigated ketamine as an anesthetic adjunct, administered either before or after induction [18,34]. Ghazaly et al. included three groups for comparison: ketamine, dexmedetomidine, and placebo [18]. They reported incidences of POD as follows: 15 (75%) in the placebo group vs. 1 (5%) in the dexmedetomidine group vs. 2 (10%) in the ketamine group, with a P-value < 0.001 [18]. While these differences were significant, the odds ratio for ketamine POD was 3.012 (95% CI: 1.185-9.681), P = 0.013, indicating that ketamine may exacerbate POD [18]. When assessing POCD, significant results persisted: placebo POCD was 7 (35%), dexmedetomidine POCD was 0 (0%), and ketamine POCD was 2 (10%), with a P-value of 0.006 [18]. The odds ratio for ketamine POCD was 4.501 (95% CI: 1.161-8.817), P = 0.006, further supporting the notion that ketamine negatively impacts cognitive function in the elderly [18].

Verdonk et al. included a placebo group and enrolled 241 more participants post-randomization than Ghazaly et al. [18,34]. They found that the difference in POD on day 7 was not significant: ketamine 6 (4.14%) vs. placebo 9 (6.34%), with an OR of 0.80 (95% CI: 0.26-2.47), P = 0.698 [34]. POCD was assessed on both day 7 and day 90. On day 7, ketamine POCD was 50 (38.8%) and placebo POCD was 54 (40.9%), OR 0.92 (95% CI: 0.56-1.51), P = 0.73, and on day 90, ketamine POCD was 26 (20.8%) and placebo POCD was 23 (20.0%), OR 1.05 (95% CI: 0.56-1.97), P = 0.884 [34]. Notably, all results were deemed insignificant in their per-protocol analysis [34]. Interestingly, Ghazaly et al. utilized a ketamine dosage twice that of Verdonk et al., with the latter study encompassing a more diverse elderly patient demographic, including participants with preoperative cognitive impairment/dementia - an exclusion criterion for most studies included in this systematic review [18,34]. When examining subgroups based on preoperative cognitive scores, no significant differences were observed [34].

Yang et al. in a retrospective cohort study involving 100 participants compared the effects of ketamine, administered throughout the surgical procedure, to a combination of ketamine and etomidate [35]. Given that this study was retrospective in nature, it is important to consider the potential for selection bias. The incidence of POCD for ketamine was found to be 20.0% (10/50), while that for ketamine-etomidate was 6.0% (3/50), with a P-value < 0.05, indicating a statistically significant difference that aligns with findings from Ghazaly et al., an RCT, suggesting that ketamine may exacerbate cognitive dysfunction in the elderly [18,35].

Impact of Ketofol on Cognitive Function in Elderly Surgical Patients

Only 2 out of the 19 studies investigated ketofol, one being an RCT and the other a multicenter prospective cohort study [12,13]. Both studies utilized the CAM to assess the incidence of POD [12,13]. Tekletsadik et al. examined its use during induction; however, only 15 patients out of 220 were induced with ketofol [12]. Among these, six exhibited POD, resulting in a COR of 0.26 (95% CI: 0.19-3.20) and an AOR of 0.22 (95% CI: 0.15-1.98), P = 0.061, which was not statistically significant [12]. Conversely, Abd Ellatif et al. reported a significant finding where the incidence of POD was higher in the placebo group compared to the ketofol group (P < 0.05) [13]. Both studies highlight the need for further investigation into the cognitive effects of ketofol in elderly surgical patients, given the limited data available.

Clinical Implications

This systematic review emphasizes the need for careful consideration of anesthetic choices in elderly patients undergoing procedures. The review reveals significant variability in the impact of ketamine and propofol on cognitive function. Three of six studies comparing propofol to inhaled anesthetics depicted a significantly higher incidence of POCD in inhaled anesthetic agents [15,16,32], with a fourth study finding that the difference in incidence was insignificant; however, the days of POD significantly more in the latter cohort [31]. Specifically, using etomidate as an adjunct to either ketamine or propofol appears to reduce the risk of POCD, suggesting a potential strategy for mitigating POCD in this vulnerable population.

Remimazolam and dexmedetomidine emerged as promising alternatives to propofol, with evidence suggesting they may be associated with better cognitive outcomes. However, the limited number of studies (only one in this systematic review) investigating remimazolam necessitates wariness in drawing definitive conclusions regarding its clinical application. Dexmedetomidine consistently demonstrated a favorable profile compared to propofol, highlighting its potential utility in elderly patients.

While results from included studies indicate a preference of one anesthetic agent over another, studies with a small number of participants risk providing underpowered findings, and interpreting these should be done with care [16,18,20,26]. Similarly, statistically insignificant findings should be cautiously considered as a repeat study with a larger cohort could provide different results [19,25]. Moreover, clinical relevance should be considered especially with results that were statistically significant but had wide confidence intervals and the inclusion of one within it. For example, the propofol POD AOR in Tekletsadik et al. was 0.14 (95% CI: 0.12-2.55), signifying uncertainty in its clinical relevance [12].

Given the aging population, it is imperative that anesthetic staff consider how their decisions may effect cognitive outcomes. The risks associated with POCD, such as prolonged hospital stays, increased mortality risk, and heightened healthcare costs, emphasize the importance of optimizing anesthesia protocols.

Strengths and Limitations of the Included Studies

The studies in this review exhibit several strengths that enhance the credibility of our findings. First, including RCTs and observational studies provides a comprehensive overview of the evidence surrounding the effects of ketamine and propofol on cognitive function. Additionally, most studies employed globally recognized cognitive assessment tools, such as the MMSE and MoCA, which enhance the reliability of measured outcomes. Additionally, score quantifications regarding what qualified as cognitive dysfunction were stated in study protocols. Several studies consisting of large sample sizes improve the generalizability of findings across different surgical contexts. Moreover, the inclusion of studies that directly compared ketamine and propofol, as well as their combinations or comparisons with other anesthetics, allows for nuanced insights into their relative effects on cognitive function.

Despite these strengths, the included studies also present limitations that must be acknowledged. The high heterogeneity among the studies regarding methodologies, anesthetic protocols, and outcome measures complicates direct comparisons and synthesis of results. Additionally, many studies assessed cognitive outcomes only in the short term, with limited follow-up periods to evaluate long-term cognitive effects. Most studies focused on a surgical subspecialty, meaning that results are specific to that patient cohort. Variability in patient demographics, anesthesia protocols, comorbidities, and surgical procedures may introduce confounding factors that influence cognitive outcomes. Additionally, concurrent use of other anesthesia agents and other medications may also have influenced results. Furthermore, the limited data on newer anesthetic agents, such as remimazolam, restrict the ability to draw definitive conclusions about their efficacy and safety.

Strengths and Limitations of Our Review

This systematic review has several strengths. It followed PRISMA guidelines and conducted a thorough search across multiple databases, ensuring a broad and inclusive selection of relevant studies. The robust quality assessment of studies using established tools, such as the Cochrane RoB2 Tool and NOS scale, enhances its credibility further. Additionally, by concentrating on elderly patients, the review addresses a critical and growing area of concern in anesthesiology and geriatrics, contributing valuable insights to both fields.

However, there are limitations to our review as well. Due to the high heterogeneity among studies, a meta-analysis was not feasible, limiting the ability to quantitatively summarize study results. Pathophysiology of inhalational anesthetics and newer anesthetic agents on cognitive function was not discussed. Moreover, the exclusion of non-English studies may have resulted in the omission of relevant research published in other languages, potentially skewing our findings.

This review did not consider the cost of anesthetic agents or whether one is more readily available than another; this may influence healthcare system decisions. Moreover, we recognize that disparities in healthcare exist from country to country, even region to region. Specifically, in a low-resource context, there may be limited access to anesthetic agents, meaning that while anesthetic agents may differentially impact cognitive function, incorporation into guidelines is not as straightforward.

Future Research Directions

Future research should focus on conducting large-scale multicenter trials to explore the comparative effects of ketamine, propofol, and alternative anesthetic agents on cognitive function in diverse elderly populations. Longitudinal studies are needed to assess the long-term cognitive impacts of these anesthetics and to identify any cumulative effects from repeated exposures. Investigations into the mechanisms underlying cognitive dysfunction related to specific anesthetic agents could help provide more thorough insights.

Moreover, the exploration of adjunctive agents such as etomidate, remimazolam, and dexmedetomidine should be prioritized. These agents have demonstrated potential benefits in reducing the risk of POCD, and their roles in combination with other anesthetics could help tailor anesthesia protocols. Research should examine optimal dosing and timing of administration to maximize cognitive protection while also ensuring effective anesthesia. Additionally, understanding how patient-specific factors, such as age, baseline cognitive function, comorbidities, and American Society of Anesthesiologists (ASA) grade, may influence anesthetic choices will be essential for developing more personalized anesthetic strategies that enhance the care of elderly patients.

## Conclusions

This systematic review analyzed the effects of propofol and ketamine on cognitive function in elderly patients, revealing that while ketamine is likely associated with an increased risk of POCD, remimazolam and dexmedetomidine positively influence cognitive outcomes compared to Propofol. Higher doses of propofol correlated with increased incidence and severity of hypoactive POD, although it demonstrated a lower incidence of POCD compared to inhaled anesthetics. Additionally, the combination of ketamine and propofol (ketofol) reduced the incidence of POD compared to placebo. Given these findings, it is paramount for physicians to consider anesthetic choices in order to minimize cognitive impact, and we recommend the development of clinical guidelines based on these and future insights. Future research should focus on larger multicenter trials to validate these findings and investigate the long-term cognitive effects of various anesthetic agents, particularly the optimal use of adjunctive agents in tailoring anesthesia protocols for this vulnerable population.
